# INTEGRATING HEALTH IT INTO CLINICAL WORKFLOW FOR DEMENTIA CARE: PROVIDER AND CAREGIVER PERSPECTIVES

**DOI:** 10.1093/geroni/igz038.3360

**Published:** 2019-11-08

**Authors:** Nicole Ruggiano, Ellen Brown, Peter Clarke

**Affiliations:** 1 University of Alabama, Tuscaloosa, Alabama, United States; 2 Florida International University, Miami, Florida, United States

## Abstract

Each year, more than 5.5 million people in the U.S. are affected by Alzheimer’s disease and related dementias (AD/RD), most of whom reside within the community, receive ongoing care from family caregivers, and medically managed by primary care providers. However, AD/RD caregiving and care is often compromised and costly, with medical costs projected to increase from $203 billion in 2013 to $1.2 trillion in 2050. A significant barrier to AD/RD care relates to collecting the health information needed for clinical decision making, due to patients’ communication problems associated with AD/RD, insufficient time to collect relevant patient information from caregivers during the medical visit, and lack of communication with in-home health and support providers. To address these issues, the research team has developed CareHeroes, a multi-functional web and mobile app designed to increase the quality of communication about patient symptoms between the caregivers and providers of people with dementia. This study integrates qualitative findings from two studies evaluating CareHeroes that involved: (a) interviews and a focus group with AD/RD caregivers and (b) focus groups with clinical staff at two memory clinics. Findings indicate that caregivers want technologies that can inform clinical decision making on a regular basis while providers emphasize the use of information technologies that prepare patients and caregivers for upcoming medical appointments. Implications for practice, research, and policy are discussed.

